# Precision Medicine in Rheumatology: The Role of Biomarkers in Diagnosis and Treatment Optimization

**DOI:** 10.3390/jcm14051735

**Published:** 2025-03-04

**Authors:** Matteo Colina, Gabriele Campana

**Affiliations:** 1 Rheumatology Service, Section of Internal Medicine, Department of Medicine and Oncology, Ospedale Santa Maria della Scaletta, 40026 Imola, Italy; 2 Alma Mater Studiorum, Department of Pharmacy and Biotechnology, University of Bologna, 40126 Bologna, Italy; gabriele.campana@unibo.it

**Keywords:** biomarkers, rheumatic disease, autoimmune disorders, precision medicine, inflammation

## Abstract

Rheumatic diseases encompass a wide range of autoimmune and inflammatory disorders, including rheumatoid arthritis (RA), systemic lupus erythematosus (SLE), psoriatic arthritis (PsA), and systemic sclerosis (SSc). These conditions often result in chronic pain, disability, and reduced quality of life, with unpredictable disease courses that may lead to joint destruction, organ damage, or systemic complications. Biomarkers, defined as measurable indicators of biological processes or conditions, have the potential to transform clinical practice by improving disease diagnosis, monitoring, prognosis, and treatment decisions. While significant strides have been made in identifying and validating biomarkers in rheumatic diseases, challenges remain in their standardization, clinical utility, and integration into routine practice. This review provides an overview of the current state of biomarkers in rheumatic diseases, their roles in clinical settings, and the emerging advancements in the field.

## 1. Introduction

Rheumatology covers a broad spectrum of diseases, ranging from those that primarily affect the joints, such as osteoarthritis, gout, and inflammatory arthritis, to more complex systemic diseases, such as SLE, RA, vasculitis, and SSc, which typically involve multiple organs. These diseases can present with a variety of symptoms and progress in ways that are often difficult to predict, making their management a complex challenge [1].

A common hallmark of many rheumatic diseases is systemic inflammation, which contributes to joint destruction, tissue damage, and multi-organ complications. Additionally, autoimmunity plays a critical role in disease pathogenesis, with the presence of autoantibodies serving as key indicators for diagnosis and disease progression [2]. Over time, the immune-mediated attack leads to irreversible tissue damage, further complicating disease management [3].

**Figure 1:**
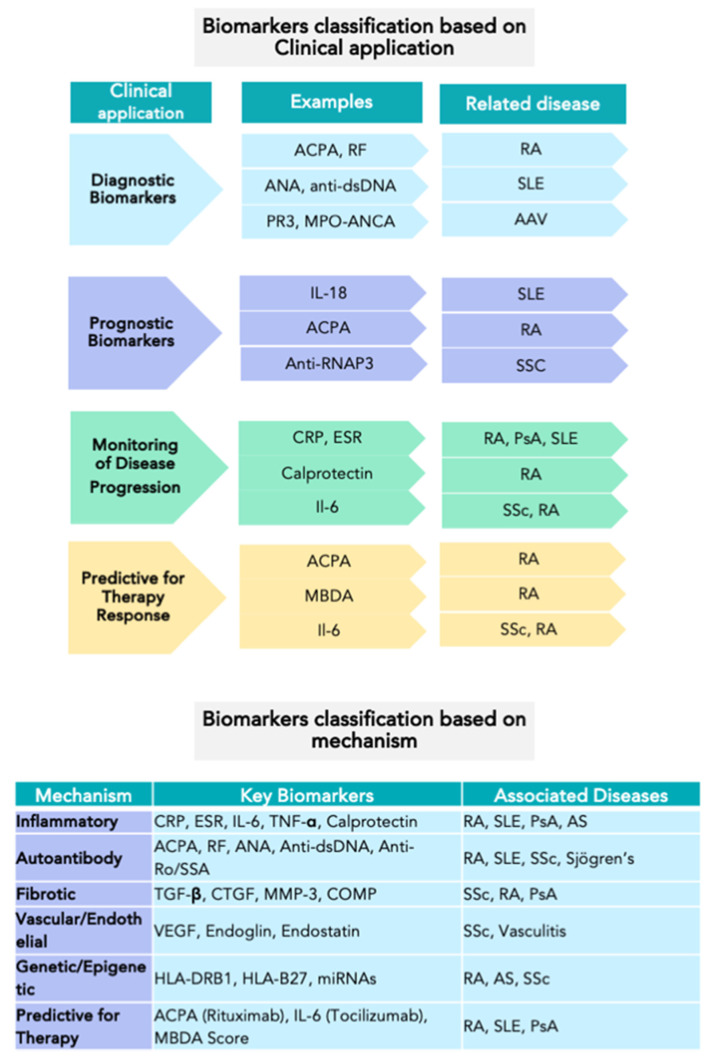
This figure provides examples of biomarkers in rheumatology classified by clinical application or mechanism. List of abbreviations: ACPA—anti-citrullinated protein antibody; RF—rheumatoid factor; ANA—antinuclear antibody; Anti-dsDNA—anti-double stranded DNA antibody; PR3-ANCA—proteinase 3 anti-neutrophil cytoplasmic antibody; MPO-ANCA—myeloperoxidase anti-neutrophil cytoplasmic antibody; IL-18—interleukin-18; Anti-RNAP3—anti-RNA polymerase III antibody; CRP—C-reactive protein; ESR—erythrocyte sedimentation rate; IL-6—interleukin-6; TNF-α—tumor necrosis factor-alpha; calprotectin—inflammatory protein complex (S100A8/S100A9); MBDA—multi-biomarker disease activity score; TGF-β—transforming growth factor beta; CTGF—connective tissue growth factor; MMP-3—matrix metalloproteinase-3; COMP—cartilage oligomeric matrix protein; VEGF—vascular endothelial growth factor; endoglin—glycoprotein (also called CD105); endostatin—fragment of type XVIII collagen; HLA-DRB1—human leukocyte antigen DR beta 1; HLA-B27—human leukocyte antigen B27; miRNAs—microRNAs; RA—rheumatoid arthritis; SLE—systemic lupus erythematosus; SSc—systemic sclerosis; AAV—ANCA-associated vasculitis; PsA—psoriatic arthritis; AS—ankylosing spondylitis.

Biomarkers have emerged as essential tools for improving disease classification, risk stratification, and personalized treatment strategies. A biomarker is defined as a measurable characteristic that serves as an indicator of normal biological processes, disease states, or therapeutic responses [4]. Biomarker Definitions Working Group defined various biomarker categories (diagnostic, disease staging, prognostic, and predictive) and emphasized their role as surrogate end-points, providing the essential guidelines for integrating biomarkers into clinical trials [5,6]. Figure 1 depicts the biomarkers based on clinical applications and mechanisms.

Biomarkers play an essential role in advancing precision medicine, offering a strategic opportunity to improve health and lower healthcare costs. Precision medicine intended as timely, personalized targeted therapies have marked a significant breakthrough in clinical practice, offering substantial improvement for managing different pathologies especially different types of cancer. However, despite the use of cutting-edge technologies, advanced bioinformatics, and machine-learning tools, similar progress has not yet been seen in the treatment of rheumatic diseases. In rheumatoid arthritis, for example, despite the availability of numerous targeted biological and traditional therapies, treatment decisions are still partially based on a process of trial and error [7].

The time for a diagnosis remains excessively long, and the primary treatment continues to rely on non-specific anti-inflammatory drugs. What makes the diagnosis of rheumatic pathologies particularly challenging are its very heterogeneous clinical presentations. Patients with similar underlying diseases can manifest with a wide range of symptoms, which often overlap with other conditions. The need for reliable, disease-specific biomarkers is more urgent than ever to make differential diagnosis easier as well as to assess disease progression and severity over time [8].

This review explores the evolving role of biomarkers in common rheumatic diseases while highlighting existing challenges in their clinical implementation.

## 2. Key Biomarkers in Common Rheumatic Diseases

This section details key biomarkers associated with rheumatic diseases, including r RA, SLE, PsA, SSc, and vasculitis, focusing on their role in diagnosis, disease severity assessment, and guiding personalized therapeutic strategies.

### 2.1. Rheumatoid Arthritis

RA is a chronic, systemic autoimmune disorder primarily affecting the small joints, causing pain, swelling, and inflammation. It is associated with progressive disability, premature mortality, and significant socioeconomic impact. RA is marked by inflammation of the synovial membranes in the joints, which results in bone erosion [9,10]. The prevalence of RA remains relatively stable, ranging between 0.5% and 1.0%, as observed in multiple European and North American populations [11].

The preclinical phase of RA, in particular the first 12 weeks after early symptoms occur, is regarded as the ideal therapeutic window to develop treatments that might ultimately prevent the disease from progressing to its clinical stages. The autoimmune mechanisms driving RA often start years before noticeable symptoms like joint pain and stiffness arise. Diagnostic biomarkers are essential tools for clinicians during this phase, as they enable the accurate identification of individuals at risk or in the preclinical stages of a condition, facilitating the use of this optimal period for intervention [9]. Beyond diagnosis, many RA biomarkers have multifaceted roles across different stages of the disease, aiding in risk stratification, monitoring disease activity, and guiding therapeutic (a summary of biomarkers discussed in this section is shown in Table 1).

#### 2.1.1. Inflammatory Biomarkers

Erythrocyte sedimentation rate (ESR) and C-reactive protein (CRP) are widely used as biomarkers in the assessment of rheumatoid arthritis (RA), reflecting the inflammatory component of the disease. The rate at which erythrocytes fall through plasma—that is, the erythrocyte sedimentation rate—indirectly indicates inflammation through increased fibrinogen and immunoglobulin levels [12].

CRP, an acute-phase reactant synthesized by the liver in response to interleukin-6 (IL-6), directly correlates with systemic inflammation and disease activity. As an acute-phase protein, the plasma concentration of CRP deviates by at least 25% during inflammatory disorders [12]. There are many factors that can alter baseline CRP levels including age, gender, smoking status, weight, lipid levels, and blood pressure [45].

Both biomarkers are integral to disease activity indices such as the 28-joint disease activity score (DAS28) and the clinical disease activity index (CDAI), aiding in monitoring disease progression, treatment response, and remission status [13].

A correlation between elevated ESR and CRP levels and radiographic as well as functional outcomes in patients with RA has been observed in several studies [14,46,47,48]. However, approximately 40% of RA patients exhibit normal ESR and CRP levels [49]. Additionally, in patients with baseline elevations, these values may remain stable despite significant clinical improvement with treatment [50].

While not disease-specific, as they can be elevated in a variety of other inflammatory and infectious conditions, as well as in malignancies, their dynamic changes provide valuable insights into the inflammatory burden in RA, complementing clinical assessments and imaging modalities and are included in 2010 ACR/EULAR Classification Criteria for RA [51].

ESR and CPR are the most widely used, but they are not the only potentially useful inflammatory markers. For instance, calprotectin has proven useful when assessing disease activity in different stages of RA [15,16].

#### 2.1.2. Autoantibodies as Biomarkers

RA is defined by the presence of autoantibodies, with rheumatoid factors (RFs) and anticitrullinated protein antibodies (ACPAs) serving as key serological markers of the disease [52,53]. Unlike autoantibodies in other autoimmune diseases, RA autoantibodies target post-translationally modified protein epitopes, collectively called antimodified protein antibodies (AMPAs). ACPAs, the most specific AMPA, has >90% specificity for RA. Other AMPAs, such as anti-CarP, AAPA, and anti-MAA, show lower specificity and are associated with inflammation and often with disease progression [21].

#### 2.1.3. Anti-Citrullinated Protein Antibodies

Citrullination is a post-translational modification of proteins that can generate novel epitopes that trigger autoantibody production [54]. ACPAs target citrullinated peptides and proteins, including keratin, fibrinogen, α-enolase, and vimentin.

ACPAs isolated from patient serum and synovial fluid are highly specific for RA, with specificity of 95% to 97% and a sensitivity of 67% to 80% [17,18]. They are present in 20–30% of RF-negative RA cases. Combining RFs and ACPAs significantly improves diagnostic accuracy, with dual positivity yielding a near 100% positive predictive value and being associated with more severe disease [19]. Via screening of a number of peptide libraries, novel citrullinated peptides were obtained and incorporated into a second-generation cyclic citrullinated protein—CCP, test (CCP2). The anti-CCP2 test is very specific for RA (95–99%) with antibodies that can be detected early in the disease. While highly specific, anti-CCP2 antibodies can occasionally be seen in other conditions, such as Sjögren’s syndrome or active tuberculosis [55].

In established diseases, high ACPA titers are linked to more severe disease progression, increased disease activity, and worse radiographic deterioration [56,57,58]. Retrospective studies show that ACPAs can be detected up to 14 years before RA symptoms, with titers rising near disease onset [59,60]. Similar findings in early RA cases confirm its value as a diagnostic and prognostic tool within the first 1–2 years of disease [18,61].

The presence of ACPA high titers can also be used to predict better responses to biologics like rituximab and abatacept, supporting its role in personalizing RA therapy [62,63,64].

Moreover, anti-CCP antibodies play a crucial role in diagnosing and predicting the progression of undifferentiated arthritis (UA), which affects 30–50% of rheumatology patients and has a variable course, with 40% progressing to RA within three years [65]. The presence of ACPAs at baseline has a higher predictive value for UA progressing to RA than other markers [66].

#### 2.1.4. Rheumatoid Factor (RF)

RFs are autoantibodies targeting the Fc region of immunoglobulin (Ig) G. While IgM RF is the most commonly measured subtype in clinical practice, IgA and IgG RF are also present. RFs are detected in up to 80% of RA patients [20], but are not highly specific due to their presence in various other conditions characterized by chronic antigenic stimulation. These include other rheumatologic disorders (e.g., systemic lupus erythematosus, Sjögren’s syndrome), some infections and malignancies, and to some degree even in healthy individuals [67].

#### 2.1.5. Other Antibody Biomarkers

Beyond well-established biomarkers such as ACPAs and RFs, several other antibodies have emerged as significant contributors to the understanding and management of RA. Among these, anti-CarP antibodies have been identified in both ACPA-positive and ACPA-negative RA patients, showing an association with more severe disease [68]. While anti-CarP antibodies exhibit lower sensitivity for diagnosing RA compared to ACPA and RF [69], the combination of RF, ACPAs, and anti-CarP antibodies in a triple-positive autoantibody profile has shown to be useful in identifying individuals at increased risk of developing RA [70].

Anti-mutated citrullinated vimentin (anti-MCV) antibodies exhibit diagnostic accuracy comparable to RFs and ACPAs, positioning them as valuable markers for RA diagnosis Additionally, elevated anti-MCV levels have been associated with the presence of extra-articular manifestations and have shown a good predictive value of disease activity at 24 months in early RA [24,25,26]. A study showed that Anti-MCV also predicts responses to rituximab [27].

Additional antibodies of growing clinical interest target peptidylarginine deiminase (PAD) enzymes, which catalyze the process of citrullination. Among these, anti-PAD4 antibodies exhibit high specificity but relatively low sensitivity [28]. Anti-PAD4 antibodies are also associated with disease severity [29].

Joint-derived Serum 14-3-3η protein is emerging as a promising biomarker for RA. When combined with RFs and ACPAs, it increases diagnostic accuracy. In addition, elevated serum 14-3-3η was relevant to more serious joint erosion and worse therapy outcomes [22,23].

#### 2.1.6. Anti-Mitochondrial Antibodies

Anti-mitochondrial antibodies (AMAs) have emerged as potential biomarkers in rheumatoid arthritis (RA). Recent research highlights their role in stratifying patients by disease phenotype and predicting erosive progression, including in seronegative cases. These findings suggest AMAs could enhance precision in RA diagnosis and prognosis [71].

#### 2.1.7. The Multi-Biomarker Disease Activity (MBDA)

MBDA is an algorithm created in an effort to obtain an objective disease monitoring system that can effectively contribute to personalized therapy. MBDA test is a tool that evaluates RA inflammation by measuring 12 serum protein biomarkers, including IL-6, TNF receptor type 1 (TNFR1), vascular cell adhesion molecule-1 (VCAM-1), epidermal growth factor (EGF), vascular endothelial growth factor A (VEGF-A), YKL-40, matrix metalloproteinases (MMP-1 and MMP-3), CRP, serum amyloid A (SAA), leptin, and resistin [72]. These biomarker levels are combined into a single score ranging from 0 to 100, offering a measure of disease activity. Studies suggest that the MBDA score has the potential to both monitor disease activity and predict radiographic progression [35,36,37].

Despite promising findings, such as the test’s utility in guiding decisions on continuing biological therapy in the setting of clinical remission [38] and predicting radiographic progression, its role in routine clinical practice remains controversial [19].

#### 2.1.8. Genetic Markers

RA has a genetic component, with the HLA-DRB1 gene being the strongest predictor of susceptibility and disease severity, including joint damage [30,31]. Although not yet used in routine clinical practice, identifying high-risk patients through genetic markers may enable personalized treatment [32].

Genetic variants have also been studied for their role in drug response, particularly in methotrexate and TNF inhibitors, but require further validation [33] and have been linked to better responses to abatacept (ABT), especially in patients with the HLA-DRB1*04:05 allele [34,73].

#### 2.1.9. TNF Inhibitors: Biomarkers Predicting Response to Therapy in RA

RA treatment has progressed from using non-steroidal anti-inflammatory drugs and corticosteroids to biologic (bDMARDs) and targeted synthetic DMARDs (tsDMARDs) [74]. There are different types of bDMARDs, with tumor necrosis factor inhibitors (TNFi) being the most widely used. Despite the expanded therapeutic options, 20–40% of patients fail to respond to their initial b/tsDMARD, and fewer than 50% achieve remission [75,76,77].

Biomarkers predicting responses to TNFα blockers in RA are essential to optimize treatment outcomes. While demographic factors like smoking status and high disease activity showed some predictive potential, traditional clinical parameters often lacked consistency. Blood biomarkers, such as CRP, RF, and anti-CCP antibodies, displayed mixed results, with some studies associating high levels of IgA RF or anti-CCP with poor responses [78,79,80]. A recent study utilizing an artificial intelligence-driven model combined with parameters such as IL-6, IL-2, CRP, and ESR proposed a predictive framework capable of effectively distinguishing between remitters and non-remitters. However, the findings require validation in a larger cohort to confirm their reliability. This study emphasized the significant potential of interleukins 2 and 6 in identifying patients likely to respond to therapy [75].

#### 2.1.10. Biomarkers of Joint Damage and Remodeling

Matrix metalloproteinase-3 or MMP3, produced by synovial fibroblasts and B cells [81,82], contributes to cartilage degradation and pannus invasion in RA [83,84]. It reflects inflammation and joint damage progression, providing a useful tool for monitoring treatment response and disease management [39,40]. However, its diagnostic performance is inconsistent. While it correlates with synovitis histology and disease activity better than some cytokines, it is surpassed by CRP and ACPAs for diagnostic accuracy [85,86]. Thus, MMP-3 complements existing biomarkers but is not superior in diagnosis [41].

Cartilage Oligomeric Matrix Protein (COMP) is an extracellular matrix (ECM) glycoprotein vital for collagen assembly and ECM stability. It plays a crucial role in maintaining cartilage integrity by supporting the structural organization and interaction of ECM components [87]. In RA, COMP levels are often elevated due to cartilage breakdown during joint inflammation and damage. Its release into the synovial fluid and circulation reflects cartilage turnover, making it a potential biomarker for joint degradation and disease progression [42,43]. Furthermore, it has been shown that a low serum level of COMP, a cartilage turnover marker, predicts a good response to adalimumab therapy in rheumatoid arthritis [44].

### 2.2. Systemic Lupus Erythematosus

SLE is a chronic autoimmune disease characterized by widespread inflammation and tissue damage, with the potential to affect multiple organs, including the kidneys, skin, brain, and cardiovascular system [88]. This systemic nature recognized nearly a century after the term “lupus erythematosus” was first used in the 19th century to describe skin lesions, underscores the disease’s complexity and severity. Vital organs and tissues, such as the brain, blood, and kidneys, are often affected, particularly in women of childbearing age [89].

Epidemiological studies highlight the growing burden of SLE, with incidence rates ranging from 0.3 to 31.5 cases per 100,000 individuals annually and prevalence rates exceeding 50 to 100 cases per 100,000 in some populations. Notably, these rates have shown a concerning upward trend over recent decades. This trend may partly reflect improved disease recognition and increasing survival rates, with the 10-year survival rate now approximately 70% [89,90,91].

The complex pathogenesis, heterogeneous clinical manifestations, and potential for multi-organ damage in SLE limit the ability of a single biomarker to fully reflect the overall disease state. Consequently, a combination of biomarkers or organ-specific markers is essential to provide a comprehensive assessment of disease activity and progression [92,93].

The clinical heterogeneity of SLE has necessitated the development of diagnostic criteria to enhance its identification and management. Early efforts defined 11 criteria, requiring the presence of at least 4 for a formal diagnosis [94]. Over time, these criteria have evolved to improve diagnostic precision and integrate advances in understanding the disease’s immunological mechanisms. The American College of Rheumatology (ACR) initially established a set of criteria in 1997, which were subsequently refined by the Systemic Lupus International Collaborating Clinics (SLICC) in 2012 [95] and further updated through a collaboration between EULAR and ACR in 2019 [96].

#### 2.2.1. Biomarkers in SLE

Although numerous publications discuss novel biomarkers for diagnosing and monitoring SLE [97,98,99], only a few are routinely used in clinical practice. The complexity and heterogeneity of SLE pose challenges in both its diagnosis and monitoring of disease progression [100]. This section provides a brief overview of the biomarkers most utilized in clinical settings.

#### 2.2.2. Autoantibodies

SLE is characterized by diverse autoantibodies targeting nuclear components, collectively termed antinuclear antibodies (ANAs) [101,102,103]. These ANAs, which bind to DNA, RNA, and related protein complexes, serve as markers for pathogenesis, classification, and disease activity [104,105,106]. ANAs are the most commonly used biomarkers, which are characteristic of SLE, but they are not specific because they can be observed in other autoimmune diseases, such as RA, systemic sclerosis, autoimmune hemolytic anemia [107,108,109], and can also be present in healthy controls [110]. ANA detected via indirect immunofluorescence (IIF) on HEp-2 cells is a key immunological biomarker for classifying and diagnosing SLE. ANA testing is included in the ACR-1997, SLICC-2012, and EULAR/ACR-2019 criteria, with an IIF-ANA titer of 1:80 or higher being an entry criterion under the EULAR/ACR-2019 guidelines [96]. Positive ANA results warrant further testing for antigen-specific ANAs like dsDNA, SSA (Ro60), Sm, and others.

Both IIF and enzyme-linked immunosorbent assay (ELISA) are commonly used for ANA testing, each with distinct advantages. IIF has high sensitivity due to its ability to detect multiple antibodies but is time-consuming and prone to false positives. ELISA uses purified antigens, offering high sensitivity and specificity but requires familiarity with the assay’s properties. The choice of method should consider the assay’s specificity, sensitivity, and diagnostic context [97].

#### 2.2.3. Reduced Complement

Early studies demonstrated that reduced complement C3 and C4 levels are associated with more severe SLE, including renal flares [111,112]. Baseline reductions in C3 or C4 can predict disease flares as early as two months to a year before they occur [113,114]. Furthermore, the utility of reduced complement levels in predicting and monitoring treatment response has been validated in clinical trials targeting B cells with rituximab or blocking the BAFF pathway [8,113,115,116].

#### 2.2.4. CRP and ESR

In comparison to patients with rheumatoid arthritis, those with SLE are more likely to exhibit ESR than CRP levels [117]. ESR rises in response to both lupus activity and infection, making it too non-specific to reliably differentiate between these conditions [118]. While ESR elevations are strongly associated with disease exacerbations in SLE [119], CRP levels generally do not correlate with other markers of disease activity, such as anti-double-stranded DNA antibodies and complement levels [120].

However, the ratio of ESR to CRP may offer additional diagnostic value, as it has the potential to distinguish between flare and infection in such patients more effectively than evaluating ESR or CRP levels individually [121].

#### 2.2.5. Cytokines and Chemokines

Cytokines associated with SLE activity include type I interferons, notably IFNα, as well as IL-6, IL-10, IL-15, IL-18, BAFF/BLyS, and TNF [122,123,124,125,126,127,128]. These cytokines play essential roles in the inflammatory processes and immune responses characteristic of SLE, indicating their importance in the disease’s pathophysiology and potential as biomarkers for monitoring disease activity.

Type I interferons, particularly IFNα, pose significant challenges in measurement, as conventional ELISA technology often proves inadequate [129]. Instead, the assessment has shifted towards analyzing peripheral blood mRNA signatures of type I interferons, which reflects the cytokine’s influence rather than direct measurement. Type I interferon signature is closely associated with the severity and progression of the disease [130,131,132].

IL-6 contributes to the inflammatory process and is important in SLE pathogenesis; however, the association with disease activity is not robust enough to make routine use likely [122]. IL-18 is clearly elevated in SLE [126] and correlates with disease severity [127,133,134]. TNF serves as a key pro-inflammatory cytokine, influencing multiple immune pathways in SLE. However, measuring these cytokines poses challenges, including variability in assay results, the impact of external factors, and the fluctuating nature of cytokine levels in relation to disease activity and treatment interventions [122,124]. Additionally, sTNFR2 levels, which are linked to both TNF activity and disease severity [135,136], present a promising candidate as a biomarker, even though they are not yet established for routine monitoring.

Serum or plasma chemokines, measurable via ELISA, are indirect markers of inflammation often produced in response to cytokines and have shown associations with disease activity in SLE. Chemokines such as CCL2/MCP-1, CXCL10/IP-10, and CCL19 are interferon-inducible genes contributing to a composite score for estimating interferon activity, which correlates with SLE disease activity. Previous studies have identified increased levels of CCL2 and CXCL10 in active SLE, reflecting interferon influence [137,138]. Additionally, CCL-11, CXCL13, and CXCL16 are associated with disease activity, while serum IL-8, CCL17, CXCL16, and CX3CL1 are linked specifically to active lupus nephritis [139,140].

### 2.3. Psoriatic Arthritis (PsA)

PsA is a chronic inflammatory condition that develops in up to 30% of individuals with psoriasis (PsO) and affects as much as 0.7% of the general population [141,142]. PsA is marked by inflammation in both axial and peripheral joints as well as entheses, presenting with a wide range of clinical symptoms. This variability often contributes to delays in diagnosis and treatment. If left unmanaged, PsA can result in progressive joint damage, leading to impaired function, permanent disability, reduced quality of life, and increased mortality [143,144,145].

PsA exhibits a heterogeneous clinical presentation and disease progression; however, many patients experience a severe, destructive form of arthritis that leads to significant morbidity and disability [146].

A delay in diagnosis is linked to a significantly reduced treatment response, while early intervention with immune-modulating or anti-inflammatory medications markedly improves both clinical and radiographic outcomes. This highlights the significance of a critical window of opportunity for implementing effective therapeutic interventions. In PsA, this “window of opportunity” appears to be wider than in RA, with the optimal timeframe for PsA intervention extending to less than a year, in contrast to RA, where it is limited to less than 12 weeks [147].

Comorbidities such as depression, anxiety, and chronic widespread pain are common in PsA and contribute to increased pain and poorer functional outcomes [148,149,150]. Additionally, previous studies have shown that female PsA patients tend to have higher disease activity and a poorer response to treatment compared to males [151].

Therefore, the management of PsA as for other rheumatic diseases should prioritize not only early intervention but also a personalized approach tailored to the individual patient. This includes considering comorbidities that may influence disease activity and treatment outcomes [147].

#### Biomarkers in PsA

The research agenda is currently moving forward to the identification of biomarkers that could help clinicians to identify subsets of patients requiring a more targeted therapy or strict monitoring and ideally to diagnose PsA at an earlier or preclinical stage in patients with skin manifestations but this remains a long-term goal for which data are limited. A recent systematic review found no specific diagnostic biomarkers that can distinguish PsA from PsO and other chronic inflammatory diseases [145]. Very few potential candidates are currently available to inform clinical practice regarding the diagnosis and prognosis of PsA [145].

Among those are bone and cartilage remodeling markers COMP and MMP3. Despite some promising findings, their discriminative capabilities have proven insufficient for clinical application. Meta-analyses revealed that COMP could differentiate PsA from osteoarthritis (OA) and healthy controls (HCs), but significant variability in serum levels across studies undermined its reliability [145] Methodological differences, such as the use of varying ELISA kits, contributed to these discrepancies. While COMP showed potential in distinguishing PsA from psoriasis (PsO), inconsistent results and a lack of quantitative data precluded comprehensive analysis of its efficacy. Similarly, serum MMP3 levels were significantly elevated in PsA patients compared to PsO patients but failed to differentiate PsA from HCs or OA.

Genetic biomarkers, including HLA alleles such as HLA-C06, HLA-B27, HLA-C02, and HLA-C12, demonstrated some potential, particularly in distinguishing axial and peripheral PsA phenotypes. Non-HLA genetic variants, including IL12B, NOS2, and IFIH1, have also been linked to PsA, though these findings remain preliminary. Autoantibodies such as anti-CCP, anti-LL37Carb, and anti-nuclear interferon-inducible protein 16 (IFI16) have been identified in PsA and PsO patients, and their presence has been associated with disease severity, but lack specificity [152,153,154,155,156]. While a variety of autoantibodies have been detected in PsO and PsA patients, their utility in clinical practice is limited, as autoantibody testing is generally not required for diagnosis or monitoring of disease activity [156].

The first-line biological treatment for PsA patients is the TNF inhibitor (TNFi), but approximately 40% of patients do not respond to these therapies. For these individuals, the process of finding a suitable and effective treatment can be time-consuming and challenging. Several studies have been conducted to propose potential candidate biomarkers able to predict therapeutic response to TNFi but to date, none of these molecules are used in clinical care due to lack of specificity [157].

### 2.4. Systemic Sclerosis

SSc, or scleroderma, is an autoimmune rheumatic disease associated with significant clinical heterogeneity, as well as high morbidity and mortality [158,159]. SSc remains incompletely understood and affects both the skin and internal organs. Its pathogenesis is marked by three key processes: vascular damage, primarily involving the microcirculation; immune system activation, autoimmunity, and inflammation; and fibrosis [160]. SSc is associated with significant clinical heterogeneity, as well as high morbidity and mortality [158,161,162].

An underlying genetic predisposition contributes to an imbalance between the innate and adaptive immune systems, resulting in the production of numerous cytokines, chemokines, and autoantibodies. This immune dysregulation triggers fibroblast activation, leading to the development of myofibroblasts and the accumulation of dense, inflexible connective tissue. While no curative therapy currently exists, significant advances have been achieved in addressing organ-specific complications [163].

The modified Rodnan skin score (mRSS) is the most widely used method for assessing skin involvement in SSc, despite its inherent subjectivity and limitations [158]. Attempts to develop more objective measures using imaging and device-assisted techniques have so far shown limited progress [164,165]. The mRSS remains a key tool for evaluating skin thickness and serves as the primary outcome measure in most clinical trials. Currently, SSc is formally classified into two main forms: the limited cutaneous form (lcSSc), characterized by distal skin thickening and associated with anticentromere antibodies, and the diffuse cutaneous form, marked by both distal and proximal skin thickening, often linked to anti-topoisomerase antibodies, anti-RNA polymerase III antibodies, or other antinuclear antibodies [160,166].

Microvascular changes, one of the defining features of SSc, are evaluated using nailfold capillaroscopy, a non-invasive technique that identifies early, active, and late SSc-specific patterns. These patterns are associated with disease progression, autoantibody profiles, and complications such as lung disease and pulmonary hypertension [167,168]. Biomarkers for SSc discussed in the next section are summarized in Table 2.

#### 2.4.1. Antibodies Biomarkers

The presence of serum autoantibodies targeting nuclear or nucleolar autoantigens is a defining serological feature of SSc. It is detected in over 95% of patients and is part of the ACR/EULAR 2013 classification criteria for SSc diagnoses [193]. Disease-specific autoantibodies play a crucial role in identifying distinct clinical subgroups of SSc, allowing for the stratification of patients into more homogeneous categories [159,194].

The most commonly found autoantibodies in SSc are anti-topoisomerase I (anti-Topo I), anti-centromere antibodies (ACA), and anti-RNA polymerase III (anti-RNAP3), while less frequently detected autoantibodies include members of a heterogeneous group of mutually exclusive antinuclear antibodies such as anti-Th/To, anti-fibrillarin, and anti-NOR90 [170]. While these autoantibodies are relatively specific to SSc, their individual sensitivity ranges from moderate to low. Each autoantibody is linked to a distinct spectrum of disease manifestations, aiding in predicting disease progression, the emergence of organ involvement, and individualized prognostic outcomes [169,195]. Other autoantibodies, such as those targeting PM/Scl proteins (PM/Scl-100 and PM-Scl-75), Ro52 (also known as TRIM21), or Ku, lack specificity for SSc and are also present in other systemic autoimmune diseases [159,170].

Serologic abnormalities are valuable for diagnosing SSc, but fewer data are available regarding their predictive value. A research indicates that SSc-specific antibodies can be present in patients years before the clinical diagnosis of SSc. Specifically, these antibodies are detectable in 75% of cases with SSc renal crisis and 40% of cases without renal dysfunction [171].

Although their exact pathogenetic role remains unclear, recent studies have demonstrated that immunocomplexes containing SSc-specific antibodies can activate endothelial cells and fibroblasts in vitro, suggesting a potential role in promoting pro-inflammatory and profibrotic effects [159,172].

#### 2.4.2. Fibrotic and Extracellular Matrix Biomarkers

The hallmark of SSc is the excessive accumulation of extracellular matrix components, such as collagen and fibronectin, driven largely by Transforming Growth Factor (TGF-β), a key mediator of fibrosis in SSc [196]. TGF-β is activated from its precursor form through integrin signaling and stimulates fibroblasts to differentiate into myofibroblasts, leading to further extracellular matrix production and suppression of metalloproteinase synthesis. This creates a self-perpetuating cycle of TGF-β production and fibrosis [177].

TGF-β-related genes are overexpressed in SSc skin and lung tissues, correlating with fibrosis and disease progression. However, the role of circulating TGF-β as a biomarker remains unclear due to conflicting studies. Some research shows elevated TGF-β levels in association with severe skin fibrosis and digital ulcers, while other studies indicate no significant correlation with lung involvement or overall disease severity [173,174,175]. Further investigation is needed to clarify its role as a biomarker in SSc. A recent study demonstrated that patients with SSc exhibit elevated levels of TGF-β1, the most well-researched isoform of the TGF-β family, in their serum. The correlation between these elevated levels and clinical symptoms suggests that TGF-β1 could serve as a biomarker of fibrotic and vascular involvement in SSc [176].

Platelet-Derived Growth Factor (PDGF) plays a significant role in SSc-related fibrosis. Secreted by various cells, PDGF acts as a mitogen and chemoattractant for mesenchymal cells. SSc patients show increased PDGF receptor expression in skin and bronchoalveolar lavage fluid, along with autoantibodies that activate PDGF receptors and stimulate fibroblast activity [177,178].

Connective Tissue Growth Factor (CTGF) is a key mediator of TGF-β-induced fibrosis in SSc [179]. Normally expressed at low levels, CTGF is significantly upregulated in fibroblasts stimulated by TGF-β and other factors, promoting the production of collagen 1 and fibronectin. CTGF expression is elevated in SSc skin, particularly in the epidermis, and is linked to the progression and maintenance of fibrosis, with early stages driven by TGF-β and later phases maintained by CTGF [180,181].

Vascular Endothelial Growth Factor (VEGF) is persistently upregulated in SSc, contributing to abnormal vessel morphology and vascular dysfunction [197]. Increased serum VEGF levels correlate with severe organ involvement, skin sclerosis, and pulmonary hypertension. VEGF may have protective effects against peripheral ischemia but shows varying patterns in different SSc manifestations, such as digital ulcers [182,198].

Growth Differentiation Factor 15 (GDF-15): GDF-15, part of the TGF-β superfamily, is elevated in SSc-ILD and correlates with impaired respiratory function, skin sclerosis, ILD severity, and pulmonary arterial hypertension (PAH) [199,200,201].

#### 2.4.3. Cytokines

Interleukin 6 (IL-6) is a pro-inflammatory cytokine involved in immune regulation, acute-phase reactant initiation, and hematopoiesis. It is produced by various cells, including lymphocytes, fibroblasts, and monocytes, and plays a role in T cell activation, B cell maturation, and cell differentiation. Elevated IL-6 levels are observed in several autoimmune diseases, including SSc [188].

IL-6, an inflammatory cytokine, plays a pivotal role in SSc pathology by driving collagen production, fibroblast activation, and inhibiting collagen breakdown. IL-6 activates JAK-STAT3 and MAP kinase pathways, promotes Th17 cell differentiation, and is highly expressed in the skin and serum of early dcSSc patients [188]. Elevated IL-6 correlates with severe skin involvement, ILD, poor prognosis, and lung function decline. in SSc-ILD. The anti-IL-6 receptor antibody, tocilizumab, has shown promise in preserving lung function in early SSc-ILD [202].

BAFF and APRIL, cytokines crucial for B cell survival and function, are also elevated in SSc patients. BAFF is associated with severe skin sclerosis, while APRIL correlates with pulmonary fibrosis. BAFF inhibition in mouse models reduced fibrosis and IL-6–producing effector B cells, highlighting its potential as a therapeutic target [203,204].

#### 2.4.4. Chemokines

Given their essential role in immune cell function, it is unsurprising that chemokines play a significant role in the development and progression of autoimmune diseases and may serve as valuable biomarkers for disease progression. In SSc, early studies identified CC chemokine 2 (CCL2) as a key factor in the development of tissue fibrosis. More recent research highlights the biomarker potential of additional chemokines, including CCL18, CX3CL1, and CXCL4, in SSc-associated interstitial lung disease [190,191].

#### 2.4.5. Vascular and Endothelial Markers

Vascular injury is one of the earliest clinical manifestations of systemic sclerosis (SSc). Microangiopathy, characterized by reduced capillary density and disorganized vascular architecture, results in chronic tissue hypoxia [205]. Endothelial damage contributes to vascular fibroproliferative lesions in various organs, leading to complications such as pulmonary arterial hypertension (PAH) [158] and renal crisis [206,207].

Endostatin is a fragment of type XVIII collagen that inhibits angiogenesis by blocking VEGF and bFGF activity [208]. Endostatin levels are significantly higher in SSc patients and positively correlate with ischemic manifestations [186,187].

Endoglin, a glycoprotein in the TGF-β receptor complex, plays a key role in angiogenesis and tissue remodeling [209]. Elevated soluble endoglin levels are found in SSc, particularly in patients with digital ulcers, anticentromere antibodies, or decreased lung diffusion capacity [183,184,185].

#### 2.4.6. Adhesion Molecules

Increased expression of adhesion molecules correlates with leukocyte recruitment to sites of inflammation, contributing to endothelial damage and tissue fibrosis. Circulating levels of various adhesion molecules may serve as indicators of the severity of vascular injury and tissue fibrosis in SSc [177,210]. A recent systematic review and meta-analysis suggest that specific circulating cell adhesion molecules such as ICAM-1, VCAM-1, PECAM-1, E-selectin, and P-selectin, can be helpful as biomarkers of endothelial dysfunction and atherogenesis in the assessment of cardiovascular risk in SSc patients [198].

#### 2.4.7. Micro RNA Biomarkers

The literature increasingly highlights assumptions about the epigenetic mechanisms involved in the development of SSc, with circulating microRNAs being one of the key areas of focus. MicroRNAs are short nucleotide sequences that play a role in cellular regulation [192,211]. The miR-138 and miR-27a microRNAs inhibit key pathways associated with epithelial-to-mesenchymal cell transition and subsequent fibrosis. In SSc patients, the relative expression of miR-138 and miR-27a is significantly lower compared to controls, with miR-138 showing an even further decrease in diffuse cutaneous SSc. This suggests that both miRNAs could serve as diagnostic biomarkers, with miR-138 being specifically indicative of disease severity [188,192]. These are only examples of many dysregulated miRNAs identified in SSc skin specimens and blood samples that are being investigated as potential biomarkers.

#### 2.4.8. CRP and ESR

CRP levels are elevated in approximately one-quarter of SSc patients, particularly in the early stages of the disease. Elevated CRP levels are associated with increased disease activity, greater severity, impaired pulmonary function, and reduced survival rates [188]. Although some studies suggest that elevated inflammatory markers are linked to pulmonary, cutaneous, and musculoskeletal manifestations of SSc, the role of ESR and C-reactive protein CRP in assessing disease activity in SSc remains a subject of controversy [189].

### 2.5. Biomarkers in Antineutrophil Cytoplasmic Antibody (ANCA)-Associated Vasculitis (AAV)

AAV is a prototypical example of a disease defined by the presence of a biomarker, with ANCA positivity detected in approximately 90% of affected patients [212]. AAV refers to a group of autoimmune diseases marked by inflammation of small to medium-sized blood vessels, leading to vascular destruction, tissue necrosis, and diverse systemic manifestations [213]. AAV is categorized into three groups based on clinical characteristics: granulomatosis with polyangiitis (GPA), microscopic polyangiitis (MPA), and eosinophilic GPA (EGPA) [214]. This condition is marked by the presence of proteinase 3 (PR3)-ANCA or myeloperoxidase (MPO)-ANCA in the serum. GPA is primarily linked to PR3-ANCA, whereas MPA and EGPA are mainly associated with MPO-ANCA, though they can occasionally be ANCA-negative [215].

ANCAs serve as distinctive markers that aid in the classification and diagnosis of GPA, MPA, and EGPA. The ANCA test is valuable for monitoring patients with persistently elevated ANCA levels, as those experiencing a resurgence or increase in ANCA levels are at a higher risk of relapse. However, adjusting or intensifying therapy based solely on ANCA levels is not recommended [214,215] as a rise in or persistence of ANCA during remission is only modestly predictive of future disease relapse [216]. A biomarker-guided maintenance treatment with RTX, based on monitoring ANCA levels and CD20+ B cells, has shown potential effectiveness. However, it may also increase the risk of relapses, and its clinical indications remain inconsistent [217].

Acute-phase markers such as CRP and ESR have limited utility in assessing disease activity due to their lack of specificity. Other biomarkers, including urinary soluble CD163, are being investigated for their potential role in evaluating disease activity, though they have yet to be validated for routine clinical use [218].

## 3. Biomarkers as Potential Tools in Prevention

One of the emerging questions in the field is whether biomarkers could play a role in the prevention of rheumatic diseases. Could they enable the early identification of individuals at risk, facilitating timely interventions to prevent disease onset or detect subclinical stages of rheumatic conditions?

Most studies in rheumatology investigating the potential for presymptomatic intervention have focused on RA. Significant progress has been made in understanding the preclinical phase of RA (Pre-RA) and in predicting the progression to inflammatory arthritis (IA) or RA. For most individuals who develop seropositive RA, a preclinical phase occurs during which RA-related autoantibodies, such as RFs and ACPAs, are elevated systemically before clinically apparent IA or RA emerges. This phase is marked by initial reactivity to a limited set of self-antigens and minimal systemic inflammation. Over time, immune responses expand, involving more autoantibody types (e.g., anti-CarP) and increased systemic inflammation (e.g., cytokines), ultimately leading to tissue injury and clinically apparent disease. Evidence suggests that ACPAs often precede other autoantibodies, reflecting early tolerance breakdown. Additional processes, such as altered autoantibody glycosylation and changes in T cell subsets, also occur during this period, indicating the complexity of the transition to overt RA [219].

This progress made in understanding pre-RA has enabled the initiation of prevention trials, with several already completed and others currently underway [220,221,222,223,224].

For most individuals who develop seropositive RA, a preclinical phase occurs during which RA-related autoantibodies, such as RFs and ACPAs, are elevated systemically before clinically apparent inflammatory arthritis (IA) or RA emerges [59,60,219,225,226]. This phase is marked by initial reactivity to a limited set of self-antigens and minimal systemic inflammation. Over time, immune responses expand, involving more autoantibody types (e.g., anti-CarP) [227,228] and increased systemic inflammation (e.g., cytokines) [59,219,226], ultimately leading to tissue injury and clinically apparent disease. Evidence suggests that ACPAs often precede other autoantibodies, reflecting early tolerance breakdown. Additional processes, such as altered autoantibody glycosylation [229] and changes in T cell subsets [230], also occur during this period, emphasizing the complexity of the transition to overt RA [231].

There have been numerous efforts to determine whether the progression from milder disease states to rheumatoid arthritis (RA) can be prevented. For example, a trial investigated whether methotrexate (MTX) could prevent the transition from undifferentiated arthritis (UA) to RA. A one-year course of MTX successfully delayed and, in some cases, prevented RA development in high-risk UA patients [232]. Similarly, a recent trial examined RA progression in ACPA-negative individuals with clinically suspect arthralgia, highlighting the critical importance of precise risk stratification. These studies demonstrated that treatment was most effective in patients at higher risk of developing RA [233]. Biomarkers play a pivotal role in achieving accurate risk stratification, making them indispensable tools for prevention-focused strategies.

These strategies are not confined to RA. For example, in a review from 2019, explored the possibility of identifying individuals with psoriasis who are at heightened risk of progressing to psoriatic arthritis (PsA). While recent advancements in treatments for psoriasis have led to significant clinical responses and remission in many patients, these therapeutic successes have not been mirrored in PsA. To improve PsA outcomes, strategies such as early aggressive treatment and exploring new therapeutic targets or combinations have been attempted. However, once PsA is established, the inflammatory burden may be difficult to modulate in many patients. Through biomarkers, it may be possible to predict this progression by integrating data from genetic, environmental, and immune factors. A predictive tool based on such biomarkers could provide the foundation for preventive clinical trials and targeted interventions, potentially halting or delaying the transition from psoriasis to PsA before it fully develops. This proactive approach would represent a major advancement in rheumatic disease prevention [234].

Ongoing proof-of-concept trials are exploring the prevention hypothesis, emphasizing the need for accurate risk prediction to design and interpret prevention strategies effectively. This evolving approach would herald a paradigm shift in managing RA and other autoimmune rheumatic diseases by proactively identifying at-risk states and intervening early to prevent disease onset [235].

## 4. Discussion

Biomarkers are crucial tools for diagnosing, monitoring, and personalizing the treatment of rheumatic diseases. However, a significant challenge in their clinical application is the considerable overlap of biomarkers across different autoimmune conditions. Many inflammatory and immune-related rheumatic diseases share common pathophysiological mechanisms, leading to similar biomarker profiles. While this overlap provides valuable insights into shared disease pathways, it complicates biomarker validation and clinical implementation by reducing disease specificity.

Inflammatory markers such as CRP and ESR are commonly elevated across various rheumatic diseases due to their association with systemic inflammation. However, their non-specific nature limits their diagnostic utility. Similarly, autoantibodies such as ANA, RFs, and ACPAs are detected in multiple autoimmune conditions. While ANA is a hallmark of SLE and SSc, it is also present in some patients with RA and PsA, as well as in healthy individuals. Likewise, RF, traditionally associated with RA, is also found in chronic infections and other autoimmune diseases. Pro-inflammatory cytokines such as IL-6, TNF-α, and BAFF are upregulated in multiple autoimmune diseases, making them valuable for monitoring disease activity but less reliable for differentiating between conditions.

Given these challenges, mechanistic biomarkers—those directly involved in disease pathogenesis—hold the greatest promise for guiding clinical decision-making. Because they reflect the dysregulation of molecular pathways integral to disease progression, they serve as more reliable predictive and pharmacodynamic biomarkers. However, the presence of shared biomarkers across multiple diseases complicates their ability to function as precise disease classifiers, increasing the risk of misdiagnosis or inappropriate treatment decisions.

To improve biomarker specificity and diagnostic accuracy, composite biomarker panels that integrate multiple disease-specific markers offer a promising solution. The Multi-Biomarker Disease Activity (MBDA) score in RA, for example, combines CRP, IL-6, TNF-R1, and MMP-3 to assess disease activity and predict treatment response more effectively than individual biomarkers. In SLE, a composite assessment of anti-dsDNA antibodies, complement proteins C3 and C4, and interferon-alpha signatures provides a more robust measure of disease activity. In PsA, integrating biomarkers such as MMP-3, COMP, and genetic factors like HLA-B27 and HLA-C06 improves differentiation between PsA and psoriasis without arthritis. These multi-marker approaches enhance precision by capturing disease-specific patterns rather than relying on a single, often non-specific, biomarker.

Another promising avenue for overcoming biomarker overlaps is the application of machine learning and artificial intelligence in biomarker classification. AI-driven models can analyze complex datasets, integrating multi-omics data from genomics, proteomics, and transcriptomics to identify unique disease signatures. Additionally, AI algorithms can combine clinical, imaging, and laboratory data to enhance diagnostic accuracy and predict treatment responses with greater precision. Such models have the potential to refine disease classification and improve personalized treatment strategies [236].

Beyond scientific validation, the practicality of biomarkers is paramount for their clinical application. To be of real value, biomarkers must be easily accessible and cost-effective, ensuring affordability and availability across healthcare settings [237]. They must also be stable during storage and handling to prevent variability in results across different laboratories [238]. Additionally, biomarkers should be non-invasive and patient-friendly, preferably detectable through blood or urine tests, to enhance patient comfort and compliance [239].

Despite significant advancements, biomarker development faces considerable challenges, particularly in validation and qualification. Longitudinal studies, which are essential for validating biomarker performance across different patient populations, require substantial time and financial investment. These studies often span years, making them resource-intensive and logistically complex. Many early diagnostic studies suffer from methodological limitations, such as small sample sizes and lack of disease prevalence considerations, leading to issues with generalizability and clinical reliability [240].

Despite these challenges, the future of personalized medicine in rheumatology remains promising. Advances in high-throughput technologies, such as transcriptomics, proteomics, and metabolomics, are enabling more comprehensive analyses of disease mechanisms. Additionally, AI-driven approaches are revolutionizing biomarker discovery by identifying novel diagnostic and predictive markers from complex datasets.

## 5. Conclusions

Biomarkers are revolutionizing the management of rheumatic diseases by enhancing diagnosis, prognosis, and treatment personalization. Despite significant progress, their routine clinical application remains hindered by challenges related to validation, standardization, and clinical integration. The lack of universally accepted biomarker panels and standardized methodologies limits their widespread adoption.

Future research should prioritize harmonizing biomarker assessment protocols, conducting large-scale longitudinal studies, and integrating multi-omics data with artificial intelligence-driven predictive models. The progress in biomarker discovery and validation has the potential to guide the transition from more traditional approaches to better patient stratification and more personalized therapies with a great impact on the quality and cost of patient care.

## Figures and Tables

**Table 1:** Biomarkers in RA. This table outlines key biomarkers associated with rheumatoid arthritis (RA), categorized according to their diagnostic, prognostic, monitoring, and predictive roles. While some biomarkers are well-established in clinical practice, others remain under investigation and have yet to be fully integrated into routine care.

References	Predictive	Monitoring	Prognostic	Diagnostic	Category of Biomarkers
					**Inflammatory**
[12,13,14]		✓	✓	✓	**ESR**
		✓	✓	✓	**CRP**
[15,16]		✓	✓		**Calprotectin**
					**Antibody Biomarkers**
[17,18]	Rituximab, Abatacept	✓	✓		**ACPA**
[19,20]		✓	✓	✓	**RF**
[21]		✓	✓		**Anti-CarP**
		✓	✓		**Anti-MAA**
		✓	✓		**AAPA**
[22,23]		✓	✓		**Serum-14-3-3η**
[24,25,26,27]	Rituximab	✓	✓	✓	**Anti-MCV**
[28,29]		✓	✓		**Anti-PAD4**
					**Genetic Biomarkers**
[30,31,32,33,34]	Abatacept	✓	✓	✓	**HLA-DRB1 (Shared Epitope)**
					**Multi-Biomarker Panel**
[35,36,37,38]	DMARD	✓	✓		**MBDA Score**
					**Joint Damage Biomarkers**
[39,40,41]		✓	✓		**MMP-3**
[42,43,44]	Adlimumab	✓	✓		**COMP**

**List of abbreviations**: ESR—erythrocyte sedimentation rate; CRP—C-reactive protein; ACPA—anti-citrullinated protein antibodies; RF—rheumatoid factor; anti-CarP—anti-carbamylated protein antibodies; anti-MAA—anti-malondialdehyde-acetaldehyde antibodies; AAPA—anti-acetylated protein antibodies; serum-14-3-3η—serum 14-3-3 eta protein; anti-MCV—anti-mutated citrullinated vimentin; anti-PAD4—anti-peptidyl arginine deiminase 4; HLA-DRB1—human leukocyte antigen DR beta 1; mBDA Score—multi-biomarker disease activity score; MMP-3—matrix metalloproteinase-3; COMP—cartilage oligomeric matrix protein; DMARD—disease-modifying anti-rheumatic Drug.

**Table 2:** Biomarkers in SSc. This table highlights biomarkers already in use or with high potential to be adopted in clinics for systemic sclerosis (SSc) across diagnostic, prognostic, monitoring, and predictive categories.

References	Predictive	Monitoring	Prognostic	Diagnostic	Category
					**Autoantibodies**
[159,169,170,171,172]		✓	✓	✓	**Anti-Topo I**
			✓	✓	**ACA**
				✓	**Anti-RNAP3**
					**Fibrotic**
[173,174,175,176]	Anti-fibrotic therapy		✓	✓	**TGF-β1**
[177,178]			✓		**PDGF**
[179,180,181]		✓	✓		**CTGF**
					**Vascular**
[182]	Vascular therapies	✓	✓	✓	**VEGF**
[183,184,185]		✓	✓	✓	**Endoglin (sEng)**
[186,187]		✓			**Endostatin**
					**Inflammatory**
[188,189]		✓	✓	✓	**CRP**
		✓			**ESR**
					**Chemokines**
[190,191]		✓	✓	✓	**CXCL4**
			✓		**CCL18**
					**Cytokines**
[188]	Anti-IL-6 therapy	✓	✓	✓	**IL-6**
					**MicroRNA**
[188,192]			✓	✓	**miR-138**
				✓	**miR-27a**

**List of abbreviations**: Anti-Topo I—anti-topoisomerase I antibodies; ACA—anti-centromere antibodies; anti-RNAP3—anti-RNA polymerase III antibodies; TGF-β1—transforming growth factor beta 1; PDGF—platelet-derived growth factor; CTGF—connective tissue growth factor; VEGF—vascular endothelial growth factor; endoglin (sEng)—soluble endoglin; CRP—C-reactive protein; ESR—erythrocyte sedimentation rate; CXCL4—C-X-C motif chemokine ligand 4; CCL18—C-C motif chemokine ligand 18; IL-6—interleukin 6; miR-138—microRNA-138; miR-27a—microRNA-27a.

## Data Availability

No new data were created.
